# Enhancing the nutritional profile of African sharptooth catfish (*Clarias gariepinus*) through dietary supplementation with natural minerals and probiotic *Escherichia coli* 39-SN

**DOI:** 10.14202/vetworld.2025.1517-1526

**Published:** 2025-06-15

**Authors:** Nurzhan Sarsembayeva, Gulmariya Ikramzhan, Tolkyn Abdigaliyeva, Zhumagul Kirkimbaeva, Birzhan Biyashev, Saule Sherimova, Primkul Ibragimov

**Affiliations:** 1 Department of Veterinary Sanitary Examination and Hygiene, Kazakh National Agrarian Research University, Almaty, Kazakhstan; 2 Department of Food Biotechnology, Almaty Technological University, Almaty, Kazakhstan; 3 Department of Microbiology, Kazakh National Agrarian Research University, Almaty, Republic of Kazakhstan; 4 Department of Veterinary medicine, M. Auezov South Kazakhstan university, Shymkent, Kazakhstan; 5 Department of Biological Safety, Kazakh National Agrarian Research University, Almaty, Kazakhstan

**Keywords:** amino acid profile, aquaculture, *Clarias gariepinus*, *Escherichia coli* 39-SN, feed additives, nutritional enhancement, vermiculite, zeolite

## Abstract

**Background and Aim:**

Feed additives composed of natural minerals and probiotics are increasingly explored in aquaculture to improve fish health and meat quality while reducing antibiotic dependency. Zeolite and vermiculite, due to their adsorptive and ion-exchange properties, enhance mineral bioavailability, whereas probiotics, such as *Escherichia coli* 39-SN improve digestive function and nutrient assimilation. This study aimed to evaluate the effects of dietary inclusion of zeolite or vermiculite in combination with *E. coli* 39-SN on the chemical, mineral, and amino acid composition of African sharptooth catfish (*Clarias gariepinus*).

**Materials and Methods:**

A total of 150 catfish were divided into three groups: A control group fed a standard diet, and two experimental groups receiving diets supplemented with either 5% zeolite or 5% vermiculite, each combined with 0.1% *E. coli* 39-SN. The trial lasted 87 days under controlled aquaculture conditions. Post-trial, muscle tissues were analyzed for moisture, fat, protein, ash content, energy value, mineral content (e.g., iron, zinc, and magnesium), and amino acid profiles using standardized laboratory methods.

**Results:**

Fish in the experimental groups exhibited improved nutritional profiles compared to the control. Notably, the zeolite + probiotic group showed the highest total amino acid content (34.63%) and significant increases in essential amino acids, such as leucine and phenylalanine. Vermiculite + probiotic supplementation yielded the highest fat content (0.69 g/100 g) and increased concentrations of iron and zinc. Both experimental groups demonstrated improved energy values, elevated mineral levels, and enhanced biological value of the muscle tissue. No signs of stress or cannibalism were observed, indicating good tolerance to the diets.

**Conclusion:**

The synergistic use of natural minerals and *E. coli* 39-SN significantly enhanced the chemical and nutritional quality of *C. gariepinus* muscle tissue. These findings suggest that such dietary strategies offer a promising alternative to antibiotics and can enhance the commercial value of aquaculture products. Future studies should explore dose optimization, long-term health effects, and the immunomodulatory potential of *E. coli* 39-SN in fish.

## INTRODUCTION

Aquaculture has emerged as a critical component of global food production in recent decades. According to a study by Fiorella *et al*. [1], global fish stocks per capita have increased by 2.3 times since the 1960s, indicating a significant rise in aquaculture. This expansion reflects the growing demand for protein-rich foods with minimal environmental impact. To maintain the high productivity of fisheries, innovative approaches are required, including the use of feed additives that improve the quality and nutritional value of fish.

Feed additives, including natural minerals and probiotics, play an important role in aquaculture. Studies by Hai and Fotedar [2], Wu *et al*. [3], and Shang *et al*. [4] have demonstrated that these additives can enhance fish immunity, accelerate growth, improve digestion, and reduce antibiotic use, making them a safe and effective alternative for improving aquaculture productivity. A study by Arciuch-Rutkowska *et al*. [5] observed beneficial chan-ges in growth rate, weight gain, cannibalism, and length gain of African catfish when fed diets enriched with β-glucan, sodium butyrate, and vitamins (C, A, D3, E, and K). These results were confirmed by Arciuch-Rutkowska *et al*. [6], who further explored the synergy between β-glucan, sodium butyrate, and certain vitamins, concluding that this mixture promoted the expression of immune-related genes. Nowosad *et al*. [7] similarly postulated that bee pollen as a feed additive could induce immunity and disease resistance due to its role as a prebiotic. They also reported that dietary inclusion of bee pollen at 1% positively affected growth, gut development, and gut microbiome composition. These studies confirm the effectiveness of implementing feed additives in aquaculture.

Conventionally, antibiotics have been widely used in aquaculture for disease prevention and treatment. However, regular and intensive antibiotic use has led to several negative consequences. Excessive use has contributed to microbial resistance, significantly reducing the efficacy of treatment [8]. Moreover, antibiotic residues are often found in end products and aquatic environments, raising concerns about food safety and ecological impacts [9]. Thus, there is a need for alternative strategies to maintain fish health and productivity without relying on antibiotics. Feed addi-tives, including probiotic strains and natural minerals, offer such alternatives by improving fish condition and product quality. Among these, zeolite and vermiculite are notable for their beneficial properties.

Natural minerals, such as zeolites and vermiculite are actively used in agriculture and aquaculture. Their properties – ion exchange, adsorption, and desorption – make them suitable for improving water quality and fish health. Zeolite adsorbs toxic substances, enhances digestion, and promotes growth. Studies by Danabaş and Dörücü [10] demonstrated that dietary zeolite improved survival and productivity in Nile tilapia (*Oreochromis niloticus*). Ibrahim *et al*. [11] reported that 2% zeolite/kg of feed increased weight gain and economic efficiency in production. Zeolite also reduces the effects of mycotoxins, as shown by Zahran *et al*. [12], who found lower mycotoxin residues in fish liver and muscle after dietary supplementation.

Vermiculite supports nutrient absorption, gastrointestinal homeostasis, and microbiological balance in fish intestines. It is a natural mineral resul-ting from hydrothermal decomposition of biotite, phlogopite, chlorites, and other magnesium-rich silica-tes [13]. Vermiculite comprises SiO_2_ (35–45%), MgO (20–40%), Al_2_O_3_ (7–15%), and Fe_2_ O_3_ (about 10%) [14]. It is added to animal feed and used as poultry litter. The USA imports about 58,000 tons of vermiculite annually, one-third of which supports livestock production [15]. Consigliere *et al*. [16] observed that vermiculite improves animal health and productivity and positively affects meat quality, likely due to its role in enhancing nutrient metabolism.

Probiotics are widely used in both agriculture and aquaculture as effective alternatives to antibiotics. They are studied for their capacity to improve digestion, increase feed digestibility, and protect fish against pathogens [17]. Probiotic preparations enhance disease resistance in fish both *in vitro* and *in vivo*, reduce disease frequency and duration [18], and are being increasingly adopted as alternatives to antibiotics [19, 20]. Their use reduces antibiotic dependency and prevents infections linked to intestinal pathogens [18].

ZeinEddine *et al*. [21] showed that the probiotic strain *Escherichia coli* Nissle 1917 improved growth and immune responses in Nile tilapia. Fish receiving this probiotic displayed increased growth and disease resistance, making it a promising candidate for aquaculture. Similarly, Nofouzi *et al*. [22] reported that goldfish (*Carassius auratus*) fed *E. coli* Nissle 1917 (at 10^6^–10^8^ CFU/g over 80 days) showed improved growth and immunity, including increased lysozyme activity and resistance to *Aeromonas hydrophila*.

This study is noteworthy for its innovative approach to enhancing the nutritional quality of aqua-culture species through a synergistic combination of natural minerals and probiotics. Notably, prior studies in the past 5 years have focused primarily on the sorbent capabilities of zeolite and vermiculite. However, the combined use of these minerals with probiotics as feed additives for juvenile African catfish has been scarcely investigated [23, 24]. A distinguishing feature of this study is the novel application of the probiotic strain *E. coli* 39-SN in the diet of African sharptooth catfish (*Clarias gariepinus*). Although *E. coli* is widely recognized for its gastrointestinal roles in terrestrial animals, its function as a beneficial microorganism in aquaculture is underexplored. The adaptability of this strain to mineral substrates and its influence on muscle amino acid levels make this study unique among fish feed supplement investigations [25].

Traditional research tends to assess feed additives and probiotics in isolation. Vijayaram *et al*. [26] have focused on combinations, such as prebiotics and probiotics or probiotics and vitamins. Recent work examining additive–probiotic combinations, such as the study by Elshafy *et al*. [27] on Nile tilapia, reported no significant improvements in fish metabolism.

Despite the well-documented individual benefits of natural minerals and probiotics in aquaculture, current research rarely investigates their combined application in a synergistic framework, particularly in carnivorous fish species, such as African sharptooth catfish (*C. gariepinus*). Existing studies have primarily focused on either the use of sorbents, such as zeolite and vermiculite for their detoxifying and mineralizing effects, or the role of probiotics, especially lactic acid bacteria and *Bacillus* species in improving gut health and immunity. However, few investigations have addressed the potential synergistic effects arising from the co-administration of mineral sorbents and probiotic bacterial strains on the nutritional profile and muscle composition of fish. Moreover, probiotic applications in aquaculture have largely excluded *E. coli* strains due to their traditional association with pathogenicity in terrestrial hosts. This has limited exploration into beneficial strains, such as *E. coli* 39-SN, a genetically characterized probiotic that has demonstrated significant immunomodulatory and growth-promoting effects in terrestrial animals, but whose potential in aquatic species remains virtually unstudied. In addition, there is a lack of mechanistic insight into how such probiotic–mineral combinations influence protein metabolism, amino acid assimilation, and mineral bioaccumulation in muscle tissue. These knowledge gaps hinder the development of integrated nutritional strategies that are both effective and sustainable for commercial aquaculture.

In light of these gaps, the present study aimed to evaluate the effects of dietary supplementation with two natural minerals, zeolite and vermiculite, each combined with the probiotic strain *E. coli* 39-SN, on the chemical, mineral, and amino acid composition of African sharptooth catfish (*C. gariepinus*). Specifically, this investigation sought to (1) determine the influence of these feed additives on the proximate composition of fish muscle (protein, fat, moisture, and ash), (2) assess changes in macro- and micro-mineral content (e.g., iron, magnesium, and zinc), and (3) analyze shifts in essential and non-essential amino acid concentrations. By employing a controlled experimental design and rigorous biochemical analyses, this study aims to elucidate the nutritional enhancements attributable to dual mineral–probiotic supplementation. The findings are expected to provide novel insights into feed formulation strategies that promote sustainable growth performance and meat quality in aquaculture, while potentially reducing dependence on antibiotics and synthetic additives.

## MATERIALS AND METHODS

### Ethical approval

This study was reviewed and approved by the Ethics Committee of the Kazakh National Agrarian Rese-arch University (Protocol 77 dated September 18, 2023).

### Study period and location

The study was conducted from February to May 2024 at Tengry Fish LLP, located in the Almaty region, 270 km from Almaty City.

### Experimental animals and rearing conditions

The object of the study was African sharptooth catfish (*C. gariepinus*) grown at the Tengry Fish LLP in the Almaty region, Kazakhstan. This fishery is located 270 km from Almaty City. For the experiment, three experimental groups, each comprising 50 specimens, were established using the analog-pair method, consi-dering the average weight of the fish to ensure the comparability of the data. During the experiment, the fish were kept under identical environmental conditions. The water temperature was maintained at 24.0°C ± 1.4°C, and the air temperature averaged 22.5°C ± 0.9°C, ensuring consistency with the physio-logical needs of *C. gariepinus*. Feeding was conducted twice daily, and feed amounts were adjusted based on biomass, temperature, and feed manufacturer guidelines. Throughout the experimental period, no cases of cannibalism or aggressive behavior were obser-ved in any group.

### Feed additives

As part of the experiment, the following feed additives were used: Zeolite, a natural mineral from the Changkanai deposit, was incorporated at a concentration of 5% in the feed. Vermiculite is a local product manufactured by Avenue LLP in the South Kazakhstan region; the dose of vermiculite was also 5%. The probiotic strain *E. coli* 39-SN is a genetically modified probiotic that has shown high efficiency in several studies by Hai *et al*. [2], Ibrahim, *et al*. [11] and Zahran, *et al*. [12]. Previously, this strain was studied in experiments on various animal species, including calves, piglets, and birds. The results of production tests have shown that *E. coli* 39-SN significantly reduces morbidity and improves the general condition of animals, increasing their survival rate to 100% [28]. The strain was obtained from the collection of microorganisms of the Research Institute (RI) for biological safety repub-lican state enterprise of the Ministry of Education and Science of the Republic of Kazakhstan (collection number M-46-15/E). Its dose in the feed was 0.1%. This concentration was selected based on a previous study by Biyashev *et al*. [28] on terrestrial animals (including calves, piglets, and poultry), in which a 0.1% inclusion rate of *E. coli* 39-SN demonstrated optimal effects in improving immunity and reducing morbidity.

### Experimental design and fish feeding

To conduct the experiments, three groups were formed: A control group and two experimental groups using the analog-pair method. Fifty specimens were attributed to each group, considering the average weight of the fish. The fish of the first (control) group received standard compound feed (RGM-5B) from the farm without feed additives. The fish of the second (experimental) group received standard compound feed supplemented with 5% zeolite feed additive and 0.1% probiotic strain *E. coli* 39-SN. The fish of the third (experimental) group received standard compound feed supplemented with 5% vermiculite feed additive and 0.1% probiotic strain *E. coli* 39-SN. The fish-feeding scheme is illustrated in Table 1.

**Table 1:** Fish feeding scheme

No.	Group	Feeding type	Number of fish
1	Control	MD (100%)	50
2	Experimental	MD (95%) + zeolite (5%) + *E. coli 39-SN* (0.1%)	50
3	Experimental	MD (95%) + vermiculite (5%) + *E. coli 39-SN* (0.1%)	50

MD=Main diet, *E. coli*=*Escherichia coli*

Feeding was conducted twice a day: In the morning and evening. Daily feed rates were calculated based on fish weight, water temperature, and recommendations from the feed manufacturer. The whole experiment lasted 87 days. The nutritional value and energy intensity of compound feed administered to fish are presented in Table 2.

**Table 2:** Percentage composition of basic nutrients and metabolic energy of compound feed used in feeding sharptooth catfish

Indicator (%)	Quantity
Moisture	72
Protein	48
Fat	11
Fiber	1.9
Ash	9.2
Nitrogen-free extractive substances	22.8
Phosphorus	1.5
Metabolizable energy (MJ/kg)	18.1

Biologically active substances in the compound feed are represented by vitamins in optimal amounts: A: 10.00 common units (cu)/kg; D: 1.50 cu/kg; E: 200 mg/kg; and C: 1,000 mg/kg. Daily rations were calculated following the biological needs of the object of the study, its weight, water temperature, and the table of feeding norms of the feed manufacturer. One-time feeding for the experimental groups consisted of the amount of compound feed calculated for each experimental group, based on the recommended daily diet, water temperature, average fish weight, and total fish biomass, multiplied by the number of feedings per day, with the addition of feed additives as specified in the experiment design.

A feed mixture of compound feed with feed additives was prepared immediately before feeding the fish to the experimental groups. The probiotic culture was applied by spraying a thin layer of feed with periodic stirring. The daily feeding rate was determined using the generally accepted growing technology, taking into account the fish’s weight and water temperature.

### Chemical analysis of muscle tissue

The study was conducted in the laboratory of the Food Safety RI. The moisture content of the muscles was determined according to the State Standard (GOST) 33319-2015 “Meat and meat products. Method for determining the mass fraction of moisture” by drying in a ShS-80-01-SPU drying cabinet (Smolenskoye Program Control System Special Design and Technology Bureau (SKTB SPU), Russia) at a temperature of 105°C. The fat content was determined on a Soxhlet extraction apparatus according to GOST 23042-2015 “Meat and meat products. Method for determining fat.” The mass fraction of total protein was determined according to GOST 25011-2017 “Meat and meat products. Methods for the determination of protein” by the Kjeldahl photometric method. The total ash content was determined following GOST 31727-2012 (ISO 936:1998) “Meat and meat products. Method for determining total ash content” by calcination in a muffle furnace (EKPS-10 SPU, model 4006) at a temperature of 550°C for 8 h. All chemical and bio-chemical analyses were performed in triplicate using samples from three randomly selected fish per group. All sample analyses were performed by laboratory personnel who were blinded to the group assignment to minimize potential bias during data collection and interpretation.

The energy value was calculated using the Alexandrov formula:

X = C × (F + A) × 4.1 + F × 9.3

Where X is the caloric content of meat (kcal/100 g), C is the amount of dry matter (g), F is the fat content (g), and A is the ash content (g).

### Mineral composition of muscle tissue

The iron, calcium, magnesium, sodium, and potassium content in meat was determined using the colorimetric method according to GOST 32343-2013 (ISO 6869:2000) “Determination of calcium, copper, iron, magnesium, manganese, potassium, sodium, and zinc by atomic absorption spectrometry.” Phosphorus content was determined using GOST 9794-2015 “Meat products. Methods for determining the total phosphorus content.” Iodine content was determined following GOST 31660-2012 “Food products. An inversion- voltametric method for determining the mass concentration of iodine.”

The ashing technique was used to determine the mineral content. Samples were ashed in a muffle furnace at 400°C–600°C following standardized procedures for mineral quantification. NH (CO) was used to accelerate the deoxidation process. The samples were placed in a bomb, filled with oxygen, and covered with a lid. The burning process took 3 min. Ash-containing metals in the form of oxides were treated with an HCl solution (1:1) to obtain metallic chlorides.

### Amino acid composition

The amino acid composition of meat was studied in accordance with the municipal institution regulatory document (ND MU) 04-38-2009, “Determination of proteinogenic amino acids.” We used capillary elec- trophoresis to measure the mass fraction of amino acids, utilizing the Kapel capillary electrophoresis system (Lumex, Russia).

### Data processing

The results were subjected to variational statistical processing using Microsoft Excel. Statistical analysis was performed using one-way analysis of variance, followed by Tukey’s Honest Significant Difference test to determine pairwise differences between groups. Differences were considered statistically significant at p ≥ 0.05.

## RESULTS

### Chemical composition of fish muscle

The chemical composition of fish is primarily characterized by the content of water, nitrogenous substances, fats, minerals, carbohydrates, and vitamins. These components also affect the nutritional value and taste of meat [29]. It varies according to physiological and environmental factors, such as age, gender, habitat, and feed composition [30].

Table 3 presents the results of the study of protein, fat, moisture, and ash content in the muscle tissue of fish across the control and experimental groups.

**Table 3:** Chemical composition of the muscles of sharptooth catfish when using different types of feed additives

Indicator (g/100g)	Groups (n = 10)		

	1 (control)	2 (experimental)	3 (experimental)
Mass fraction of protein	18.52 ± 0.22	18.65 ± 0.21*	17.94 ± 0.21*
Mass fraction of fat	0.42 ± 0.006	0.49 ± 0.005*	0.69 ± 0.001
Moisture	73.25 ± 0.88*	68.49 ± 0.82*	69.48 ± 0.71
Dry substances	26.75 ± 0.22	31.51 ± 0.18	30.52 ± 0.29
Ash	1.35 ± 0.02	2.11 ± 0.02*	1.26 ± 0.01*
Energy value	177.78	198.05	188.85

* p *≥* 0.05

In the control group, the mass fraction of protein was 18.62 ± 0.22 g/100 g, which was 0.4% higher than in the first experimental group (zeolite + probiotic; 18.5 ± 0.21 g/100 g, p ≥ 0.05). In the second experimental group (vermiculite + probiotic), the protein content was lower (17.94 ± 0.21 g/100 g), representing a 3.6% decrease compared with the control. These differences in protein levels between groups were small, suggesting stable protein retention under the experimental condi-tions. The decrease in group 3 may be attributed to the presence of vermiculite in the diet, which warrants fur-ther investigation.

Conversely, group 3 showed a notable increase in fat content, reaching 0.69 ± 0.001 g/100 g, 39.1% higher than the control group (0.42 ± 0.006 g/100 g) and 28.3% higher than group 1 (0.49 ± 0.005 g/100 g, p ≥ 0.05). This elevated fat content suggests that vermiculite may influence lipid metabolism, resulting in a higher energy density in the product.

Moisture content was highest in the control group (73.25 ± 0.88 g/100 g), compared to 68.49 ± 0.82 g/100 g and 69.48 ± 0.71 g/100 g in groups 1 and 2, respectively. This reduction in water content in the experimental groups may indicate that the feed additives modulate water metabolism.

Ash content was 1.35 ± 0.02 g/100 g in the control, 2.11 ± 0.02 g/100 g in group 1, and 1.26 ± 0.01 g/100 g in group 2 (p ≥ 0.05). The increase in group 1 suggests a shift in mineral retention due to the influence of zeolite and probiotics.

The energy value of meat was 177.78 kcal/100 g in the control group, corresponding to its low fat and high moisture content. Group 1 had an energy value of 198.05 kcal/100 g – an 11.3% increase – while group 2 showed 188.85 kcal/100 g, a 6.2% increase over the control and 4.6% lower than group 1. The increased caloric value in the experimental groups is attributed to elevated fat and reduced moisture contents, indicating enhanced nutrient density due to feed supplementation.

### Mineral composition of fish muscle tissue

The mineral elements in fish muscles are crucial for physiological function and nutritional quality. Major minerals include calcium, phosphorus, magnesium, sodium, potassium, zinc, and iron. Table 4 presents their concentrations across the three groups.

**Table 4:** The content of macro- and micro-elements in the muscle tissue of fish of the control and experimental groups

Elements (mg/100g)	Group (n = 10)		

	1 (control)	2 (experimental)	3 (experimental)
Potassium	264.86 ± 3.18	270.04 ± 3.24*	263.28 ± 3.16*
Calcium	42.76 ± 0.51*	41.49 ± 0.50*	42.63 ± 0.50
Magnesium	23.07 ± 0.27	24.56 ± 0.29	23.01 ± 0.27
Sodium	61.88 ± 0.74	61.23 ± 0.72*	62.15 ± 0.75
Phosphorus	187.21 ± 2.25	181.67 ± 2.18*	188.16 ± 2.26*
Iron	1.18 ± 0.01	1.25 ± 0.01	1.29 ± 0.02*
Zinc	0.52 ± 0.006*	0.56 ± 0.007	0.61 ± 0.007
Iodine	0.006 ± 0.0001	0.006 ± 0.0001	0.007 ± 0.0001

p ≥ 0.05

In the control group, the potassium concentration was 264.86 ± 3.18 mg/100 g (p ≥ 0.05), which was 2.2% lower than in group 1 (270.04 ± 3.24 mg/100 g) and 0.6% higher than in group 2 (263.28 ± 3.16 mg/100 g). Calcium was 42.76 ± 0.51 mg/100 g in the control, 3.1% higher than group 1 (41.49 ± 0.50 mg/100 g), and 2.1% lower than group 2 (42.63 ± 0.50 mg/100 g).

Magnesium content was 23.07 ± 0.27 mg/100 g in the control, 24.56 ± 0.29 mg/100 g in group 1, and 23.01 ± 0.27 mg/100 g in group 2. Sodium was 61.88 ± 0.74 mg/100 g in the control, decreased slightly in group 1 (61.23 ± 0.72 mg/100 g), and increased in group 2 (62.15 ± 0.75 mg/100 g). Phosphorus content was 187.21 ± 2.25 mg/100 g in the control, 181.67 ± 2.18 mg/100 g in group 1, and 188.16 ± 2.26 mg/100 g in group 2 (p ≥ 0.05).

Iron levels increased from 1.18 ± 0.01 mg/100 g in the control to 1.25 ± 0.01 mg/100 g in group 1 (5.9% increase), and further to 1.29 ± 0.02 mg/100 g in group 2 (9.3% increase). Zinc rose from 0.52 ± 0.006 mg/100 g in the control to 0.56 ± 0.007 mg/100 g in group 1 (7.7% increase), and 0.61 ± 0.007 mg/100 g in group 2 (17.3% increase).

These results demonstrate that feed additives containing zeolite, vermiculite, and *E. coli* 39-SN positively influenced the mineral profile of fish meat. Enhancements were particularly notable in potassium, iron, and zinc, while calcium, magnesium, sodium, and iodine remained relatively stable.

### Amino acid composition of muscle tissue

This study investigated how effect of combining zeolite or vermiculite with probiotic strains on the levels of essential amino acids (EA) and non-essential amino acids (NEA) in *C. gariepinus* muscle tissue. Table 5 provides detailed data from the 87-day feeding trial.

**Table 5:** Amino acid composition of sharptooth catfish when using various feed additives

Amino acid (%)	Groups (n = 10)		

	1 (control)	2 (experimental)	3 (experimental)
Arginine	1.97 ± 0.78	2.45 ± 0.78*	2.16 ± 0.23*
Histidine	0.13 ± 0.06	1.65 ± 0.82	0.33 ± 0.12
Leucine + isoleucine	1.75 ± 0.45	3.96 ± 0.03	2.89 ± 0.14
Lysine	2.50 ± 0.85	2.46 ± 0.17	2.35 ± 0.11
Methionine	0.84 ± 0.28*	1.15 ± 0.39*	0.86 ± 0.23*
Phenylalanine	1.97 ± 0.59	2.97 ± 0.89	1.92 ± 0.52
Threonine	1.18 ± 0.47	2.64 ± 0.05*	2.18 ± 0.12*
Valine	1.47 ± 0.58	2.79 ± 0.51*	1.25 ± 0.62
ΣEA	11.81 ± 4.06	20.07 ± 3.64*	16.12 ± 2.09
Alanine	1.45 ± 0.37	3.13 ± 0.81	2.47 ± 0.36
Aspartic acid	2.14 ± 0.88*	2.08 ± 0.15*	2.35 ± 0.12
Glutamic acid	0.96 ± 0.25	2.29 ± 0.12*	1.36 ± 0.22
Glycine	1.05 ± 0.36	1.79 ± 0.29	1.95 ± 0.62*
Serine	1.02 ± 0.26	3.46 ± 0.90*	0.92 ± 0.11
Tyrosine	0.95 ± 0.28	1.81 ± 0.54	0.98 ± 0.27
ΣNEA	7.57 ± 2.40	14.56 ± 2.81	10.03 ± 1.70
EA/NEA	1.56	1.37	1.60
Total amount of amino acids	19.38	34.63	26.15

* p *≥* 0.05, ΣEA is the amount of EA, ΣNEA is the amount of NEA, EA=Essential amino acids, NEA=Non-essential amino acids

The highest arginine content was recorded in group 2 (2.45%), possibly due to its role in nitrogen compound synthesis and anabolic activation from zeolite and probiotics (p ≥ 0.05). Histidine content significantly increased in group 2 (1.65%) compared to group 3 (0.33%) and the control (0.13%). The sum of these amino acids reached 3.96% in group 2, greatly exceeding the control (1.75%). Lysine levels were also elevated in both experimental groups, though differences were not statistically significant.

Methionine increased slightly in both group 2 (1.15%) and group 3 (0.86%) compared to the control (0.84%) (p ≥ 0.05). Group 2 also showed a notable increase in phenylalanine (2.97%) and alanine (3.13%), reflecting enhanced metabolism of aromatic amino acids and gluconeogenic activity.

The sum of essential amino acids (ΣEA) was highest in group 2 (20.07%), nearly double that of the control (11.81%). Group 3 reached a 16.12% (p ≥ 0.05) rate. Key increases involved leucine, isoleucine, threonine, and phenylalanine, which were particularly elevated in group 2 (3.96%) compared to the control (1.75%).

**Figure 1:**
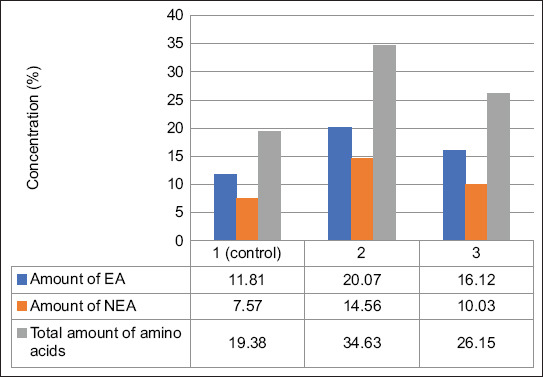
Concentration of EA and NEA. EA=Essential, NEA=Non-essential amino acids.

The non-essential amino acid content also increased significantly in the experimental groups. Group 2 recorded 14.56%, followed by Group 3 (10.03%), both of which exceeded the control (7.57%). Alanine and serine increased in group 2 indicate enhanced energy metabolism and synthetic activity. Alanine rose from 1.45% in the control to 3.13% in group 2 (Figure 1).

The EA/NEA ratio was 1.56 in the control – the highest among all groups. In group 2, the ratio dropped to 1.37, indicating a greater relative increase in NEA. Group 3 had a ratio of 1.60, similar to the control, suggesting a balanced additive effect on metabolism.

Total amino acid content in the control group was 19.38%. This rose to 34.63% in group 2 (the highest overall), and 26.15% in group 3. These increases can be attributed to the improved protein metabolism and amino acid assimilation promoted by zeolite, vermiculite, and *E. coli* 39-SN, which collectively enhanced digestive efficiency and nutrient uptake in muscle tissue.

## DISCUSSION

### Protein dynamics and probiotic modulation

Our study revealed notable changes in the proportions of protein, fat, moisture, and ash in the muscle tissue of fish. Proteins are biologically the most important and chemically complex substances necessary for the formation of organs and tissues. The growth and development of the body are impossible without their participation [31], as they also contribute to maintaining energy balance and forming structural components [32]. This aligns with recent studies by Riaz *et al*. [33] and Totewad and Gyananath [34] on the effects of probiotics on freshwater fish, such as *Cirrhinus mrigala* and *Cyprinus carpio*, which sho-wed improvements in fat, ash, protein, and moisture content.

Due to the limited number of studies examining *E. coli* as a probiotic in aquaculture, its potential can only be inferred from data derived from terrestrial animal models [35]. In our study, the protein content in experimental group 2 (zeolite + probiotic) increased slightly to 18.65 g/100 g, whereas it decreased slightly in group 3 (vermiculite + probiotic). The lower protein content in the vermiculite group may be attributed to the relatively weaker sorption capacity of vermiculite compared to zeolite, which may limit its influence on protein metabolism. Similar outcomes were observed by Biswas *et al*. [36], who demonstrated increased protein and lipid content in *O. niloticus* when probiotics were administered, with untreated fish exhibiting higher moisture and ash content. Abdelhamid *et al*. [37] also reported increased muscle protein content in shar-ptooth catfish following probiotic supplementation.

However, these findings contrast with studies by Elshafy *et al*. [27] and Sheikhzadeh *et al*. [38], who reported no significant metabolic improvements from zeolite supplementation in fish diets.

### Lipid accumulation and sensory quality

Lipids in fish play a key role in energy metabolism and significantly influence sensory characteristics [39]. Fat content directly affects taste and nutritional value, with higher fat associated with improved palat-ability [40]. In our study, fat content was lowest in the control group (0.42 g/100 g), increased modestly in group 2 (0.49 g/100 g), and was highest in group 3 (0.69 g/100 g). Zeolite may enhance lipid metabolism, while vermiculite appears to have a more pronounced effect, potentially by improving lipid bioavailability or reducing oxidative degradation.

This contrasts with Elshafy *et al*. [27], who report- ed decreased fat levels in Nile tilapia supplemented with zeolite and probiotics. Meanwhile, limited data exist on vermiculite’s use as a feed additive; for instance, Apdraim *et al*. [41] found no significant changes in poultry fat levels with vermiculite supplementation.

### Enhancement of energy value and marketability

The energy value of fish is influenced by its content of healthy fats, proteins, and, to a lesser extent, carbohydrates [42]. The control group exhibited an energy value of 177.78 kcal/100 g. In group 2, this rose to 198.05 kcal/100 g, while group 3 showed 188.85 kcal/100 g. These increases correlate with higher fat and protein content in the experimental groups, supporting the hypothesis that dietary additives enhance meta-bolic processes, promoting nutrient assimilation and deposition.

For aquaculture, increasing energy value improves the nutritional quality and market value of fish.

### Influence of feed additives on mineral bioavailability

Mineral composition is known to vary based on species, age, diet, and environmental factors [43]. Our study observed elevated potassium (+2.2%), mag-nesium (+6.5%), iron (+5.9%), and zinc (+7.7%) in the experimental groups compared to the control, indic-ating a positive effect of the feed additives on mineral bioaccumulation.

This finding is consistent with those of Nssar [44] and Zenhom *et al*. [45], who reported enhanced tissue mineral concentrations and increased fish produ-ctivity following zeolite supplementation. These results confirm the beneficial effects of natural minerals and probiotics on enhancing the micronutrient profile of fish meat.

### Amino acid enhancement and metabolic efficiency

The amino acid composition was also significantly enhanced in the experimental groups, particularly in group 2 (zeolite + probiotic), which exhibited the highest total amino acid concentration. These results support previous research by Wang and Ji [46] and Shadieva *et al*. [47], which demonstrate that probiotics, especially when combined with mineral supplements, can improve protein metabolism and amino acid absorption.

This improvement may result from increased digestive efficiency, nutrient bioavailability, and overall metabolic activation. Given the novelty of using *E. coli* 39-SN in aquaculture, additional research is necessary to assess its probiotic efficacy in aquatic species. Kirkimbayeva *et al*. [48] highlighted the immunological and microbial modulation potential of this strain in fish.

## CONCLUSION

This study demonstrated the positive effects of dietary supplementation with natural minerals, including zeolite and vermiculite, combined with the probiotic strain *E. coli* 39-SN on the nutritional composition of African sharptooth catfish (*C. gariepinus*). The findings revealed that the combination of zeolite and probiotics (Group 2) significantly enhanced protein retention (18.65 g/100 g), increased essential amino acid concentrations (ΣEA = 20.07%), and improved mineral profiles, particularly for iron, zinc, and magnesium. Vermiculite supplementation (Group 3) showed the most pronounced increase in fat content (0.69 g/100 g) and energy value (188.85 kcal/100 g), highlighting its potential to improve lipid metabolism.

These results highlight the potential of using mineral–probiotic combinations in aquafeeds to enha-nce muscle quality, nutrient density, and energy content in cultured fish. Such enhancements are crucial for increasing consumer acceptance, market value, and the sustainability of aquaculture production systems. In addition, the use of *E. coli* 39-SN as a novel probiotic agent offers an alternative strategy for reducing anti-biotic dependency in fish farming, addressing growing concerns over antimicrobial resistance and residue accumulation.

The study is distinguished by its integrated approach, which combines natural sorbents with a genetically characterized probiotic strain not previously applied in aquaculture. The rigorous analysis of protein, lipid, mineral, and amino acid profiles, conducted using standardized protocols and a blinded evaluation of outcomes, enhances the validity and reproducibility of the findings.

The research was conducted on a single fish species under controlled farm conditions and did not include assessments of immunological or gut microbiota. In addition, the long-term effects of *E. coli* 39-SN on fish health and its environmental impact remain unexplored.

Further studies should investigate the molecular and immunomodulatory effects of *E. coli* 39-SN in diverse aquaculture species. Comparative trials invol-ving different probiotic strains, mineral ratios, and dosing strategies are recommended to optimize feed formulations. Assessments of intestinal histomorphol-ogy, oxidative stress biomarkers, and microbiome composition will provide deeper mechanistic insights.

The incorporation of zeolite or vermiculite in combination with *E. coli* 39-SN into fish diets offers a promising strategy for enhancing the nutritional profile and market quality of *C. gariepinus*. This mineral–probiotic synergy may contribute to the development of more efficient, health-oriented, and sustainable aquaculture systems.

## DATA AVAILABILITY

All the generated data are included in the manuscript.

## AUTHORS’ CONTRIBUTIONS

NS: Conceptualization, methodology, and writing – original draft preparation. GI: Data curation, investigation, and writing – review and editing. TA: Formal analysis, visualization, and writing – original draft. ZK: Laboratory analysis, validation, and writing – review and editing. BB: Resources, project administration, and supervision. SS: Software, data curation, and statistical analysis. PI: Funding acquisition, supervision, and writing – review and editing. All authors have read, reviewed, and approved the final manuscript.
